# An extended thermal pressure equation of state for sodium fluoride

**DOI:** 10.1107/S1600576725000330

**Published:** 2025-02-01

**Authors:** Lewis A. Clough, Nicholas P. Funnell, Christopher J. Ridley, Dominik Daisenberger, Joseph A. Hriljac, Matic Lozinšek, Ross J. Angel, Simon Parsons

**Affiliations:** a EaStCHEM School of Chemistry and Centre for Science at Extreme Conditions University of Edinburgh King’s Buildings, W. Mains Road EdinburghEH9 3FJ United Kingdom; b ISIS Neutron and Muon Source Rutherford Appleton Laboratory Didcot OxfordshireOX11 0QX United Kingdom; c Neutron Scattering Division Oak Ridge National Laboratory Oak Ridge TN37831 USA; d Diamond Light Source, Harwell Science and Innovation Campus, Didcot, OxfordshireOX11 0DE, United Kingdom; e Jožef Stefan Institute Jamova 39 1000 Ljubljana Slovenia; f Istituto di Geoscienze e Georisorse Consiglio Nazionale delle Ricerche (CNR) Corso Stati Uniti 4 35127 Padova Italy; The University of Western Australia, Australia

**Keywords:** sodium fluoride, equation of state, bulk modulus, thermal expansion

## Abstract

The effect of pressure and temperature on the unit-cell volume of NaF has been measured by X-ray powder diffraction at ambient pressure between 12 and 300 K and neutron powder diffraction up to 5 GPa between 140 and 350 K. These data have been combined with high-pressure volume data at 300 and 950 K to 25 GPa and adiabatic bulk modulus data to 650 K to define an equation of state for NaF relating molar volume to both temperature and pressure. The model combines a fourth-order Birch–Murnaghan equation of state at 295 K with a Mie–Grüneisen–Debye model for thermal pressure. The parameters of the model set at 295 K and ambient pressure are as follows: reference unit-cell volume *V*_0_ = 14.9724 (5) cm^3^ mol^−1^, isothermal bulk modulus *K*_0*T*_ = 46.79 (14) GPa, first derivative of the bulk modulus *K*′_0*T*_ = 5.72 (12), second derivative of the bulk modulus *K*′′_0*T*_ = −0.43 (4) GPa^−1^, Debye temperature *T*_MGD_ = 459 (3) K, and Anderson Grüneisen parameters γ_0_ = 1.547 (11) and *q* = 0.94 (18).

## Abstract 2

A *PVT* equation of state has been determined for sodium fluoride with a range of validity between 12 and 950 K in temperature and 0 and 25 GPa in pressure. This equation of state is based on a fourth-order Birch–Murnaghan expression for isothermal compression and a Mie–Grüneisen–Debye model for thermal pressure.

## 1. Introduction

Application of high pressure is an increasingly common method for studying the mechanical properties of materials (McKellar & Moggach, 2015 ▸; Moggach & Oswald, 2020 ▸). In large-volume clamps the pressure can be determined resistively using materials such as manganin, but in most smaller-volume crystallographic and spectroscopic experiments it is usually measured by loading a standard for which the mechanical or spectroscopic properties are known as a function of pressure along with the sample in the cell used to apply load. In diamond anvil cell work, where optical access is possible, the fluorescence line shift of ruby is very commonly used to determine the pressure at room temperature (*e.g.* Rekhi *et al.*, 1999 ▸; Shen *et al.*, 2020 ▸). The ruby scale has been extended to temperatures up to 700 K for pressures up to 120 GPa (Wei *et al.*, 2011 ▸). The Raman shift of the diamond anvils themselves can also be used to measure pressure, but tends to be applicable only at pressures of above 10 GPa (Zouboulis *et al.*, 1998 ▸; Akahama & Kawamura, 2010 ▸).

In cases where a sample is sensitive to the laser radiation or when the sample chamber is not optically accessible, a diffraction standard can be used by employing a known equation of state (EoS). Some common standards are quartz (Angel *et al.*, 1997 ▸; Scheidl *et al.*, 2016 ▸), gold (Matsui, 2010 ▸), lead (Fortes, 2019 ▸) and NaCl (Decker, 1965 ▸; Brown, 1999 ▸). A small quantity of one of these materials can be included in a sample chamber and its lattice parameters can be used to infer the pressure experienced by the sample at an independently measured temperature. Gasket materials such as rhenium (Xian *et al.*, 2022 ▸) and tungsten (Kozyrev & Gordeev, 2023 ▸) can also be measured simultaneously with the sample if no other options are available, though the gaskets are often subject to highly non-hydro­static stresses (Kondrat’yev & Vohra, 2007 ▸) and are very stiff, meaning that uncertainties in the pressures are relatively large.

The number of materials for which a *PVT* EoS has been parameterized, relating volume to both pressure and temperature, is nevertheless quite limited, particularly at low temperature, and the aim of this paper is to define a suitable set of parameters for sodium fluoride. NaF offers some unique benefits as a pressure marker: it is non-reactive towards oxidizing or fluorinating agents, it is insoluble in common hydro­static media, and it is relatively soft and therefore suitable for use at pressures below 10 GPa.

NaF has the NaCl structure type in the space group *Fm*3*m*, with the approximate lattice parameter *a* = 4.63 Å and unit-cell volume *V* = 99.3 Å^3^ under ambient conditions. It remains stable in this phase over a wide range of temperatures from 0 K to its melting point of 1266 K at ambient pressure, and up to 28 GPa at 300 K. Bridgman (1931 ▸) determined the compressibility of NaF at both 300 and 348 K, corresponding to a bulk modulus of 48.3 (6) GPa at 300 K. Yagi (1978 ▸) and Sato-Sorensen (1983 ▸) provided thermal expansion and bulk modulus data between 300 and 1073 K, obtaining bulk moduli of 45 (1) and 46 (6) GPa, respectively. A study by Liu *et al.* (2007 ▸) reports volumes to almost 1000 K and 25 GPa, yielding a bulk modulus of 46 (1) GPa at 300 K. Additionally, several elasticity studies to determine the adiabatic bulk modulus (*K*_*S*_) of NaF have been performed (Haussuhl, 1960 ▸; Miller & Smith, 1964 ▸; James & Yates, 1965 ▸; Lewis *et al.*, 1967 ▸; Bensch, 1972 ▸; Jones, 1976 ▸). The majority of these were carried out at ambient or elevated temperatures (up to 650 K) and yielded a *K*_*S*_ value at room temperature and pressure between 44.7 and 48.5 GPa. Of these studies, only those of Lewis *et al.* (1967 ▸) and James & Yates (1965 ▸) extend to temperatures below room temperature. Low-temperature data, which would be beneficial for precise determination of characteristic temperatures, are thus very limited; therefore, we have measured the thermal expansion of NaF between 12 and 295 K and its compressibility in the ranges 0–5 GPa and 140–350 K, using these data in combination with the selected literature data to determine a new *PVT* EoS for NaF.

## 2. Experimental

### 2.1. Source of materials

NaF was obtained in polycrystalline form (Thermo Scientific ≥99% ACS reagent grade). The sample was thoroughly dried under vacuum at 333 K for 5−6 h before use. Halocarbon oil 11-14, which was used as a hydro­static medium, was obtained from Halocarbon Product Corporation and used as supplied; the label on the stock is available in Fig. S1 of the supporting information. The properties of this medium have been described in more detail by Motaln *et al.* (2025 ▸).

### 2.2. Data collection procedure

Variable-temperature X-ray powder diffraction data were collected using Cu *K*α_1_ radiation on a Rigaku SmartLab instrument equipped with an Oxford Cryosystems PheniX cryostat operating between 12 and 300 K. The instrument was calibrated using a NIST silicon standard, and the temperature uncertainty is 0.2 K.

Variable-pressure and variable-temperature neutron powder diffraction data were obtained using the time-of-flight technique on the PEARL instrument at the ISIS neutron and muon source (Bull *et al.*, 2016 ▸). Approximately 60 mm^3^ of polycrystalline NaF was loaded into a null-scattering Ti–Zr alloy capsule gasket (shown in Fig. S2) for a V3B-type Paris–Edinburgh (PE) press along with a lead pellet as a reference pressure marker (Besson *et al.*, 1992 ▸; Besson & Nelmes, 1995 ▸; Fortes, 2019 ▸). Halocarbon oil was used as a pressure-transmitting medium. The temperature was varied between 140 and 350 K in increments of 30 K and monitored using two K-type thermocouples buried in the anvils of the press. Previous tests have shown that they represent the sample temperature to within 0.5 K. The temperature fluctuation recorded during each data collection was within ±1 K. Pressure was monitored and adjusted by means of a computer-controlled hydraulic system and data were measured at load increments of 5 tonnes (∼0.5 GPa) up to a maximum load of 50 tonnes (∼5 GPa). Although the applied load was constant during each temperature scan, the pressure experienced by the sample varied as the temperature cooled due to contraction of the gasket and freezing of the pressure-transmitting medium.

Diffraction data suitable for unit-cell parameter and structure refinement were measured over the *d-*spacing range 0.5–4.1 Å. The intensity scale of the summed pattern was normalized with respect to the incident-beam monitor and the scattering from a standard vanadium calibration sample. The intensities were further corrected for the wavelength and scattering-angle dependence of the neutron attenuation by the PE press anvils (constructed from zirconia and alumina) and gasket (Ti–Zr) materials (Marshall & Francis, 2002 ▸; Funnell *et al.*, 2021 ▸).

### 2.3. Data processing and fitting

All structure refinements were carried out using *TOPAS-ACADEMIC* (Coelho, 2018 ▸). The X-ray powder data collected at ambient pressure between 12 and 300 K were modelled using Rietveld refinement (Rietveld, 1969 ▸). The positional parameters of the Na^+^ and F^−^ ions are fixed by symmetry; thermal motion is likewise constrained to be isotropic. The instrumental line-shape contribution was defined using a fundamental parameters model (Cheary & Coelho, 1992 ▸) and the sample contribution using the TCHZ peak function. The data obtained will be referred to below as the *VT* dataset.

Neutron powder data were also modelled with the Rietveld method, including contributions from NaF, the pressure marker (Pb) and the anvil materials (alumina and zirconia). Sample contributions to peak shapes were modelled with pseudo-Voigt functions and the thermal parameters for all components were modelled at the isotropic level. Pressures and their standard uncertainties were calculated during fitting using the unit-cell volume of Pb and EoS parameters derived by Fortes (2019 ▸). The data obtained will be referred to as the *PVT* dataset. The refined line-width parameters of NaF and Pb were 407 (6) and 361 (11) at 0.336 (5) GPa and 290 K and 510 (11) and 406 (17) at 4.79 (6) GPa and 140 K, suggesting that the effect of non-hydro­staticity was modest in the range of applied conditions. Since both lead and NaF are cubic, small deviatoric stresses will result in the same relationship between volume and mean normal stress as the hydro­static EoS.

The refinements yielded the unit-cell volume (*V*) of NaF as a function of pressure (*P*) and temperature (*T*), which were fitted to a Mie–Grüneisen–Debye (MGD) EoS (Anderson, 1995 ▸) using a combination of *EoSFit7_GUI* (Gonzalez-Platas *et al.*, 2016 ▸) for variable-temperature and variable-pressure data and *EoSFit7c* (Angel *et al.*, 2014 ▸) for scaling of multiple datasets and subsequent *PVT* fitting. Refinement weights were the inverse variances of the volumes and pressures obtained from the Rietveld refinements. Data plotting was performed using the *Seaborn* library in Python (Waskom, 2021 ▸). A table containing all data used for fitting is provided in Table S1 of the supporting information.

## 3. Results

### 3.1. Equation of state and modelling

The relationship between pressure and volume was modelled using Birch–Murnaghan (BM) EoSs (Birch, 1947 ▸). These are based on the assumption that the excess free energy of compression can be expressed as a power series in Eulerian finite strain *f*_E_ [equation (1)], a function of the experimentally measured unit-cell volume *V* and the volume *V*_0_ under reference conditions (taken as ambient pressure):
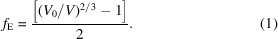
The power series can be truncated to second, third or fourth order to yield systematically more elaborate descriptions of compression behaviour. At fourth order the pressure (*P*) is parameterized in terms of *V*, *V*_0_, the isothermal bulk modulus *K*_0*T*_ at the reference pressure, and its first and second derivatives with respect to pressure (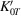 and 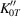):
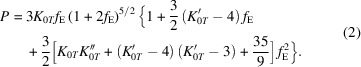
The term in 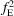 in equation (2) is zero in the third-order BM EoS; the second-order form is further obtained by setting 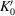
= 4.

The effects of temperature *T* were modelled using thermal pressure Δ*P*_th_, the pressure required to suppress the effects of thermal expansion along an isochor [equation (3), where *T*_0_ is a reference temperature, 295 K in this work, and 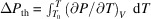]:
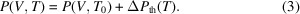
Thermal pressure arises through the effects of phonons, which may be treated using Einstein, Debye or Kieffer oscillator models. A Debye oscillator is used in the MGD formulation (Angel *et al.*, 2018 ▸), yielding equation (4),
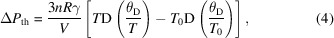
where D(θ_D_/*T*) is the Debye third-order function, θ_D_ is the Debye characteristic temperature, *n* is the number of atoms per formula unit and *R* is the gas constant. γ is the Grüneisen parameter [equation (5)], which reflects the variation in vibrational frequencies ω with *V*. The thermal expansion coefficient α does not appear explicitly in equation (4); it may be accessed through the Grüneisen relationship [equation (5)] between α, the isothermal bulk modulus *K*_*T*_ and the constant volume heat capacity *C_V_*, all at a given set of *P*–*T* conditions:
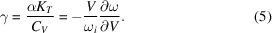
In the quasi-harmonic approximation, the value of γ is constant along an isochor, but it was assumed by Anderson (1995 ▸) to vary with *V* from a reference value γ_0_ at a volume *V*_0_ according to equation (6):
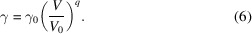
The value of the parameter *q* in equation (6) is usually found to be near 1 (Boehler & Ramakrishnan, 1980 ▸; Boehler, 1982 ▸), and it may be refined along with γ_0_, *V*_0_, the bulk modulus and its derivatives, and the Debye temperature during EoS fitting. An alternative ‘*q* compromise’ approach is to assume that θ_D_ and the ratio γ/*V* both remain constant. Though these two assumptions contradict one another, the approach is useful in refinements when data quantity is more limited as it removes a refinable parameter (*q*) from the model (Kroll *et al.*, 2019 ▸; Angel *et al.*, 2020 ▸).

The use of the Debye oscillator in the MGD thermal pressure model implies that phonon dispersion is accounted for in an approximate way but, unlike some other approaches, it also ensures that the thermal expansion is reduced to zero at 0 K. In addition, the parameters γ_0_ and *q* directly imply the Debye temperature(s) of the phonons driving thermal expansion, meaning that experiments in which *V* is determined as a function of both *T* and *P* provide constraints on these parameters in addition to those gained from inclusion of adiabatic bulk modulus data.

### 3.2. EoS modelling

After initial fitting of individual datasets to obtain estimates of parameters, the BM (to third order) and MGD models were refined together using the *VT* and the complete *PVT* sets of volume data. All parameters (including *q*) were refined, along with a scale factor for the volumes in the *PVT* dataset relative to those in the *VT* dataset (Ehlers *et al.*, 2022 ▸). The resulting parameters are given in Table S2.

The parameters listed in Table S2 were obtained within ranges of temperature and pressure (12–350 K and 0–5 GPa) that are relatively narrow by comparison with other studies on NaF (Yagi, 1978 ▸; Sato-Sorensen, 1983 ▸; Liu *et al.*, 2007 ▸). Of these, Liu *et al.* (2007 ▸) obtained powder diffraction data along the 300 K isotherm and between 718 and 989 K up to pressures of 25 GPa. The volume data listed in their paper were combined with those obtained here in order to obtain a more broadly applicable EoS.

**Figure 1:**
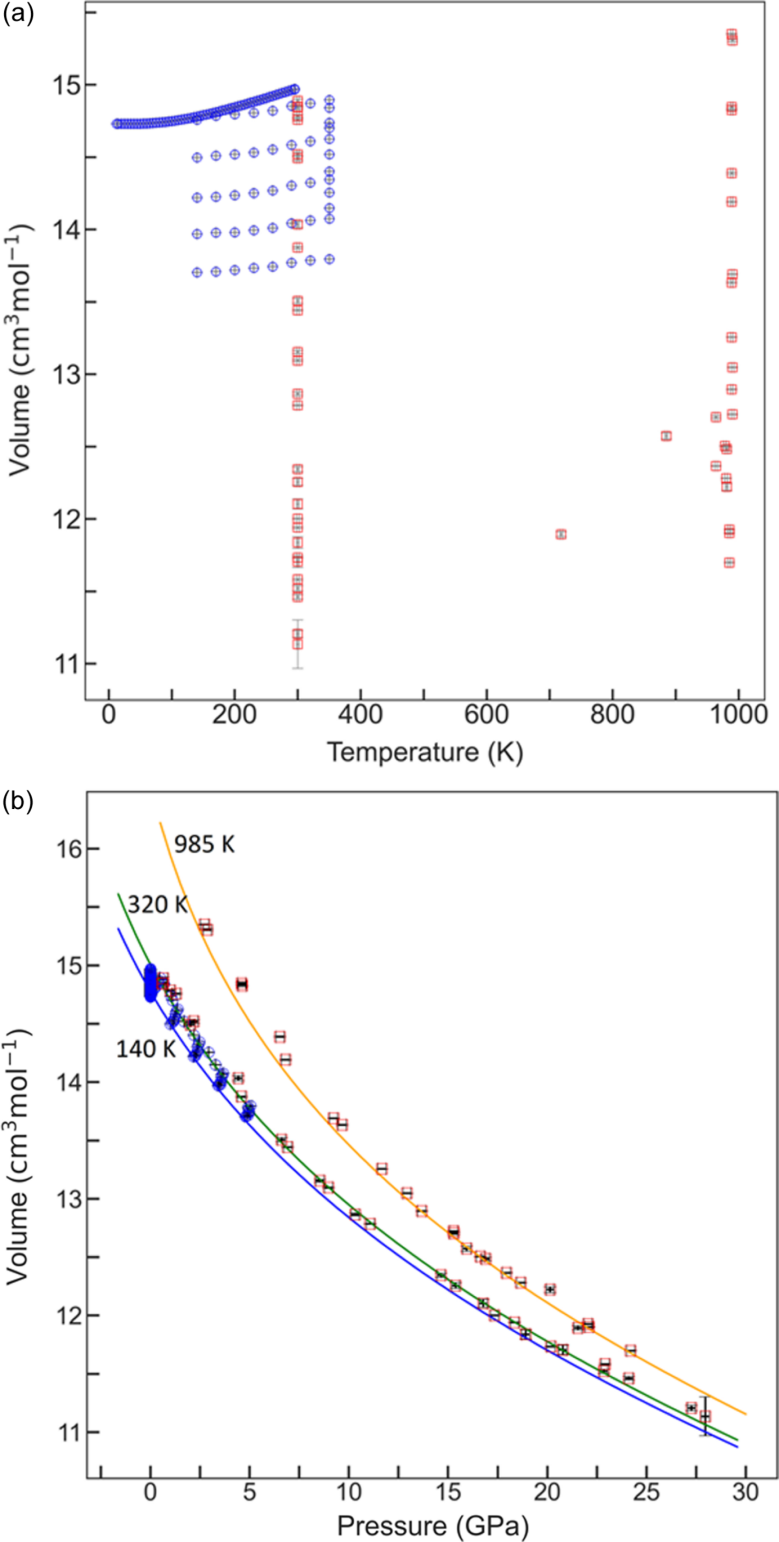
(*a*) Temperature–volume coverage obtained in this study (blue circles) in combination with data obtained by Liu (2007 ▸) (red squares). (*b*) Pressure–volume coverage obtained in this study in combination with that of Liu (2007 ▸) along with selected isotherms at 140, 320 and 985 K calculated from the EoS model parameters listed in Table 1 ▸.

Although fits using third-order BM models are adequate for both our data and Liu’s data when fitted separately, extension to fourth order significantly improves the fitting statistics for the combined dataset for both *q* refined and *q* compromise models. The 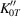 value for the fourth-order fit is significantly different from that implied at third order. The value of *K*_0*T*_ decreases slightly, while that of its pressure derivative increases. The value of θ_D_ is primarily constrained by the *VT* data and the value changes very little. With a total of 156 data points, refinements of *q* in both a *q* compromise and a *q* refined form were carried out, and the fits were compared. The refined parameters for all models are shown in the Table S3. The high quantity and quality of the combined datasets enables refinement of *q* to within a reasonable uncertainty. By refining this value, a better overall fit to the data is obtained. The overall coverage of the data obtained by this study and those of Liu *et al.* (2007 ▸) are shown in Figs. 1 ▸(*a*) and 1 ▸(*b*). Selected final fitted isotherms from the final EoS (see below) are also shown in Fig. 1 ▸(*b*).

The adiabatic bulk moduli (*K_S_*) measurements from elasticity studies at a range of temperatures (Haussuhl, 1960 ▸; Miller & Smith, 1964 ▸; Lewis *et al.*, 1967 ▸; Bensch, 1972 ▸; Jones, 1976 ▸) are an additional source of external data. Of these studies, Lewis *et al.* (1967 ▸) provides *K*_*S*_ at 4.3 and 300 K, and Jones (1976 ▸) provides equations to calculate the values they obtained between 300 and 650 K. The other authors do not provide raw data, and thus their data cannot be included in this study, though their values for *K*_*S*_ range from 44.7 to 48.5 GPa at 300 K. The model obtained from the parameters listed in Table S3 predicts a *K*_*S*_ value of 48.04 GPa at 300 K.

**Figure 2:**
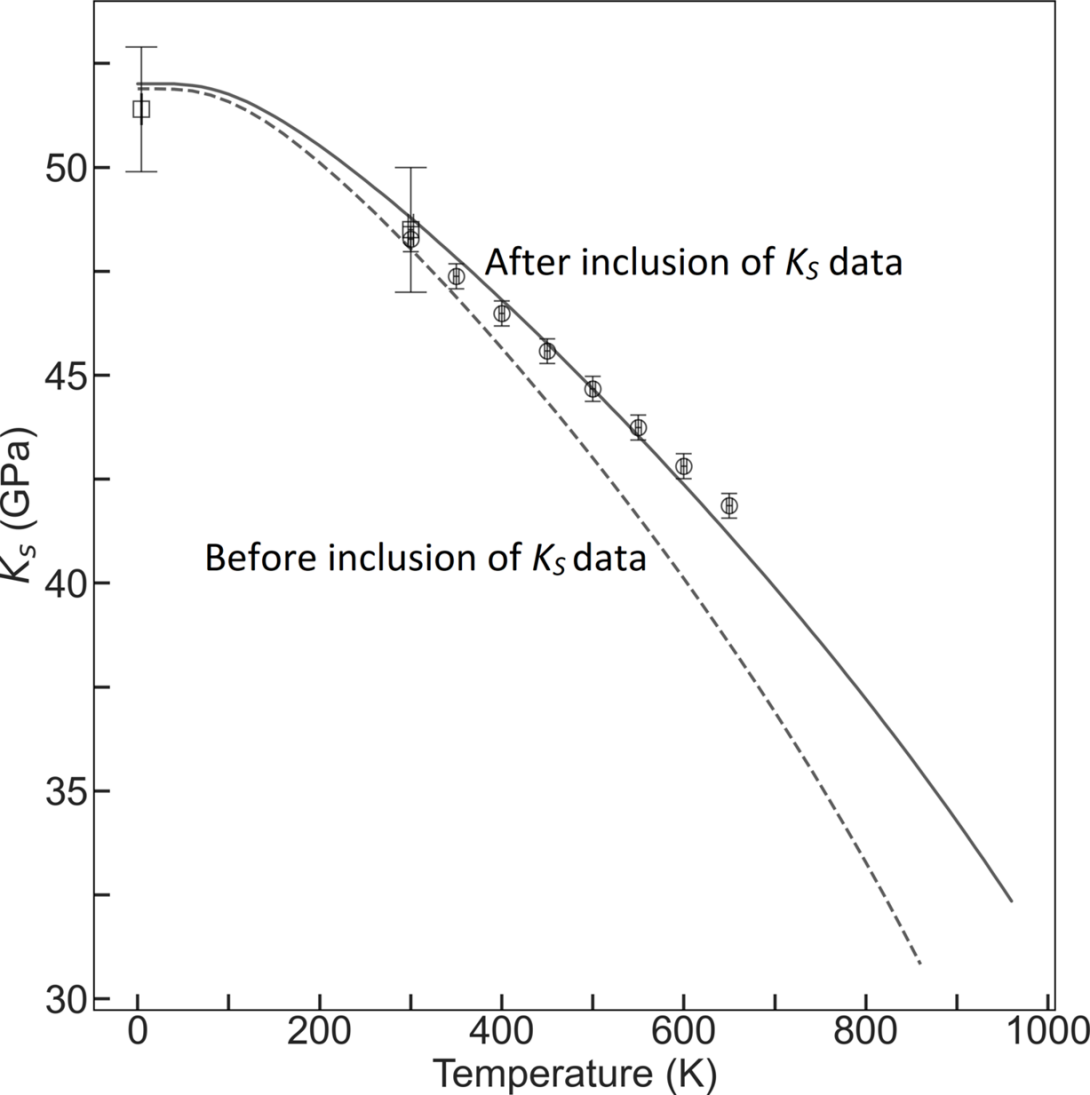
*K*_*S*_ data from Lewis *et al.* (1967 ▸) and Jones (1976 ▸) compared with values predicted by the model obtained by a combination of data from *PV* data alone (dashed line) and after inclusion of the *K*_*S*_ values in fitting (solid line).

Whilst there is agreement between our calculated *K*_*S*_ value and those measured in previous studies at 300 K, the divergence increases at higher temperatures (Fig. 2 ▸) because the volume data do not independently constrain the value of *K*_*S*_ and its pressure derivatives at high temperatures. The *K*_*S*_ elasticity data were therefore combined with our data and Liu’s volume data in order to apply a further constraint on the EoS model at high temperatures. No scaling was applied to the elasticity data. The addition of the *K*_*S*_ data clearly improves the quality of fit to the high-temperature data whilst retaining the quality of fit for the low-temperature data points (Figs. 1 ▸ and 2 ▸).

The final EoS parameters are listed in Table 1 ▸; a parameter file suitable for use in *EoSFit* that also includes the full variance–covariance matrix of the refined parameters is included in the supporting information.

## 4. Discussion

The *PVT* EoS for NaF obtained in this work is based on the combination of a fourth-order BM EoS and an MGD model for thermal pressure with reference conditions of ambient pressure and 295 K. It was obtained using variable-pressure and variable-temperature volume data from 0 to 5 GPa and 140 to 350 K, and variable-temperature volume data collected at ambient pressure between 12 and 300 K measured as part of this work, combined with volume data reaching 989 K and 25 GPa and adiabatic bulk modulus data reaching 650 K, which are available in the literature. The addition of the adiabatic bulk moduli increased the precision of *K*_0*T*_ by a factor of over 3. The final dataset, which comprises 156 volume data points and ten *K*_*S*_ data points, is the largest yet used to define the EoS of NaF. The extent of the dataset enables precise estimates of pressure to be obtained from measured unit-cell volumes. For example, if the volume of NaF is known to a precision of 0.02 cm^3^ mol^−1^, then the pressure can be calculated with an uncertainty of 0.06 GPa, assuming a temperature uncertainty of ±1 K. The range of validity is approximately 0–1000 K and 0–25 GPa.

The value obtained for the isothermal bulk modulus, 46.79 (14) GPa at 295 K, is close to those found by Sato-Sorensen (1983 ▸), Yagi (1978 ▸), Liu (2007 ▸) and Bridgman (1931 ▸) (averaging 45.7 GPa), though with a significantly smaller uncertainty as a result of the more extensive dataset. The implied thermal expansion coefficient at 295 K and atmospheric pressure is 9.79 (3) × 10^−5^ K^−1^, giving a value that is similar to though slightly lower than that found by Deshpande (1961 ▸) of 9.939 (9) × 10^−5^ K^−1^.

The γ_0_ value obtained [1.547 (11)] is very close to the value of 1.55 predicted by James & Yates (1965 ▸) from heat capacity and compressibility measurements. The refined value of *q* [0.94 (18)] is within the range anticipated for similar solids and minerals (approximately 1). Application of the *q* compromise model increases θ_D_ marginally from 459 (3) to 474 (4) K but otherwise does not change the EoS parameters significantly.

The data in Table 1 ▸ differ from the values of θ_D_ [488 (2) K versus 459 (3) K] and γ_0_ (0.91 and 1.02 versus 1.547) derived by Birch *et al.* (1979 ▸), but this may reflect the very narrow range of temperature (2.03–20.05 K) used in that study. The Debye temperature in Table 1 ▸ corresponds to a vibrational frequency of 9.56 THz (319 cm^−1^), a value which is near the frequency at the maximum of the phonon density of states of NaF (Messaoudi *et al.*, 2015 ▸), the slightly higher value likely reflecting the effects of dispersion.

**Figure 3:**
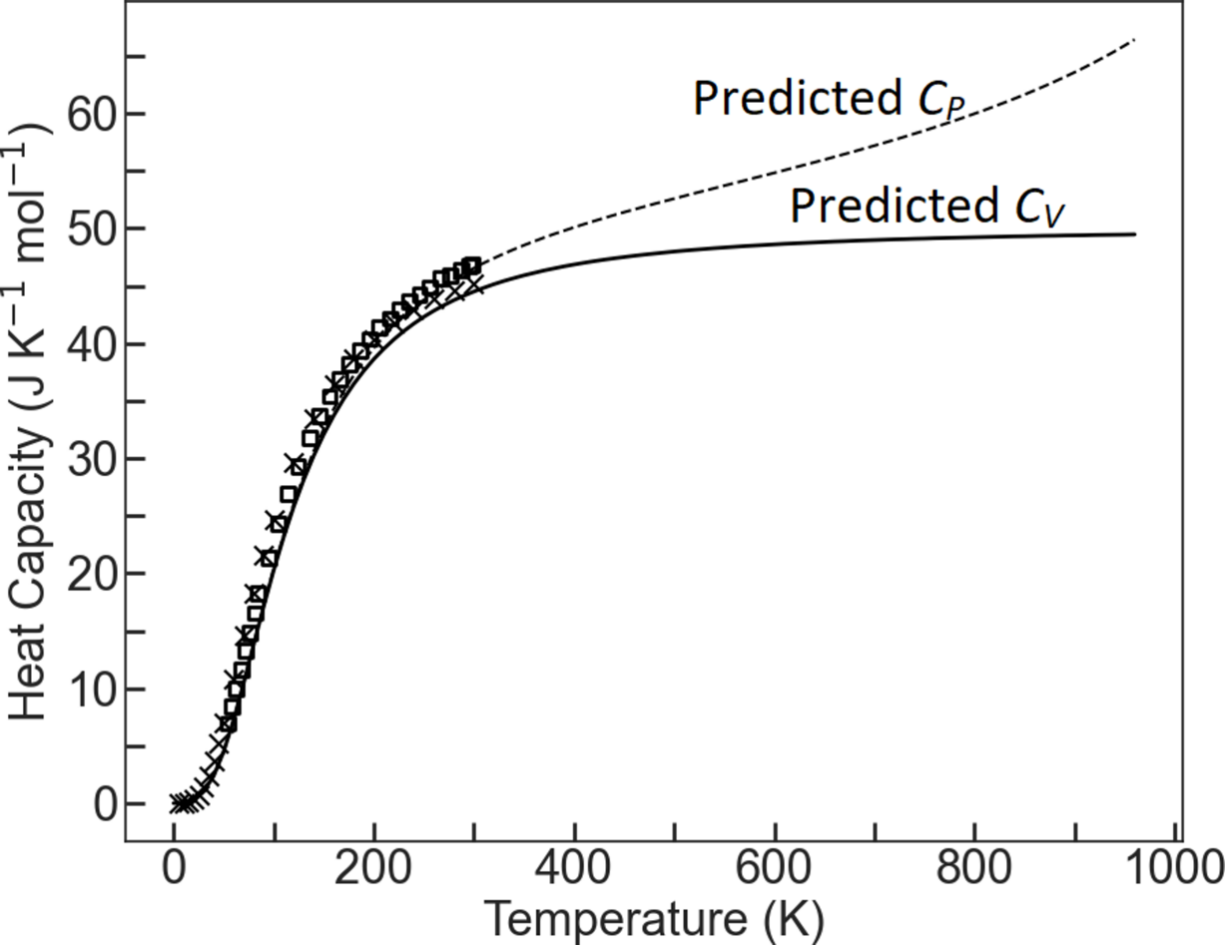
*C_P_* and *C_V_* values from King (1957 ▸) and Karo (1959 ▸) shown as squares and crosses, respectively, compared with those predicted by our final EoS shown as dashed and solid lines.

Selected isotherms from the final model are compared with the data measured in this study in Fig. 1 ▸(*b*), while the heat capacity at constant pressure (*C_P_*) and at constant volume (*C_V_*) can be used as a source of independent verification. The *C_P_* of NaF has been measured experimentally by King (1957 ▸), and Karo (1959 ▸) predicted the value of *C_V_* at various temperatures from a phonon model based on the Born lattice theory. *C_P_* and *C_V_* values between 0 and 980 K can also be predicted from the EoS model given in Table 1 ▸. The comparison between these values is shown in Fig. 3 ▸. Evidently, despite the simplicity of the single phonon model employed in this EoS, there is a remarkably good match between the calculated and experimental data.

## Supplementary Material

Supporting tables and figures. DOI: 10.1107/S1600576725000330/oc5042sup1.pdf

.eos file for PVT calculations with EoSFIT software. DOI: 10.1107/S1600576725000330/oc5042sup2.txt

Volume and adiabatic bulk modulus data in ascii format. DOI: 10.1107/S1600576725000330/oc5042sup3.txt

## Figures and Tables

**Table 1:** Recommended EoS parameters for NaF

295 K parameters	Fourth-order fit with *q* refined
*V*_0_ (cm^3^ mol^−1^)	14.9724 (5)
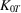 (GPa)	46.79 (14)
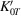	5.72 (12)
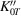 (GPa^−1^)	−0.43 (4)
θ_D_ (K)	459 (3)
γ_0_	1.547 (11)
*q*	0.94 (18)
*W*–*χ^2^*	2.73
Implied α at 295 K (K^−1^)	9.79 (3) × 10^−5^
Scale factors	1 (*VT* dataset)
	0.99964 (8) (*PVT* dataset)
	1.0034 (11) [*PVT* data from Liu *et al.* (2007 ▸)]
	1 [elasticity data from Jones (1976 ▸)]
	1 [elasticity data from Lewis *et al.* (1967 ▸)]

## Data Availability

The supporting information includes tables of all data used in fitting and the values of parameters at various stages of data inclusion. The final eos file produced in this study, from which *P* can be calculated for given values of *V* and *T*, is included along with the volume and *K*_*S*_ data points listed in Table S1 in a suitable format for direct input into *EoSFit*. Neutron diffraction data are available at https://doi.org/10.5286/ISIS.E.RB2310292.
