# Supplementary material for: An extended thermal pressure equation of state for sodium fluoride
Source: J Appl Crystallogr. 2025 Feb 1;58(Pt 1):227–32. doi: 10.1107/S1600576725000330 (PMC11798522; doi:10.1107/S1600576725000330)
Supplement: Supplementary file 1 [file j-58-00227-sup1.pdf]

JOURNAL OF APPLIED CRYSTALLOGRAPHY

Volume 58 (2025)

Supporting information for article:

An extended thermal pressure equation of state for sodium fluoride

Lewis A. Clough, Nicholas P. Funnell, Christopher J. Ridley, Dominik Daisenberger, Joseph A. Hriljac, Matic Lozinšek, Ross J. Angel and Simon Parsons

Electronic Supporting information

An extended thermal pressure equation of state for sodium fluoride (NaF).

Authors

Lewis A. Clough[a], Nicholas P. Funnell[b], Christopher J. Ridley[bc], Dominik Daisenberger[d], Joseph A. Hriljac[d], Matic Lozinšek[e], Ross J. Angel[f]* and Simon Parsons[a]*

[a]EaStCHEM School of Chemistry and Centre for Science at Extreme Conditions, University of Edinburgh, King's Buildings, W. Mains Road, Edinburgh, EH9 3FJ, United Kingdom

[b]ISIS Neutron and Muon Source, Rutherford Appleton Laboratory, Didcot, Oxfordshire, OX11 0QX, United Kingdom

[c]Neutron Scattering Division, Oak Ridge National Laboratory, Oak Ridge, Tennessee, 37831, United States

[d]Diamond Light Source, Harwell Science and Innovation Campus, Didcot, Oxfordshire, OX11 0DE, United Kingdom

[e] Jožef Stefan Institute, Jamova 39, 1000 Ljubljana, Slovenia

[f]Istituto di Geoscienze e Georisorse, Consiglio Nazionale delle Ricerche (CNR), Corso Stati Uniti 4, 35127 Padova, Italy

Correspondence email: rossjohnangel@gmail.com; s.parsons@ed.ac.uk

Synopsis A *PVT* equation of state has been determined for sodium fluoride with a range of validity between 12 and 950 K in temperature and 0 and 25 GPa in pressure based on a fourth order Birch-Murnaghan expression for isothermal compression and a Mie-Grüneisen-Debye model for thermal pressure.

Abstract The effect of pressure and temperature on the unit cell volume of NaF has been measured by X-ray powder diffraction at ambient pressure between 12 and 300 K and neutron powder diffraction up to 5 GPa between 140 and 350 K. These data have been combined with high-pressure volume data at 300 K and 950 K to 25 GPa and adiabatic bulk modulus data to 650 K to define an equation of state for NaF relating molar volume to both temperature and pressure. The model combines a fourth order Birch-Murnaghan equation of state at 295 K with a Mie-Grüneisen-Debye model for thermal pressure. The parameters of the model referred to 295 K and ambient pressure are: reference unit cell volume V_0 = 14.9724(5) cm^3 mol^{-1}, isothermal bulk modulus K_{0T} = 46.79(14) GPa, first derivative of bulk

IMPORTANT: this document contains embedded data - to preserve data integrity, please ensure where possible that the IUCr Word tools (available from http://journals.iucr.org/services/docxtemplate/) are installed when editing this document.

modulus $K_{0T}^{'}$ = 5.72(12) GPa^{-1}, second derivative of bulk modulus $K_{0T}^{''}$ = −0.43(4), Debye temperature T_{MGD} = 459(3) K, Anderson Grüneisen parameters γ = 1.547(11), q = 0.94(18).

Contents

Table S1	Table of data used for fitting.	3-6
Table S2	EoS parameters determined from our data alone.	7
Table S3	EoS parameters determined from the combination of our and Liu *et al.*'s data.	7
Table S4	EoS parameters derived from q-compromise and q-refined models.	8
Figure S1	Batch details of the halocarbon oil used as a pressure transmitting medium.	9
Figure S2	The Paris-Edinburgh cell and the TiZr gasket used for neutron diffraction.	10

Table S1 Unit cell volume data obtained in this study and by Liu *et al.* (2007) used for fitting of the equation of state. Adiabatic bulk modulus data of Lewis *et al.* (1967) and Jones (1976) are also listed.

T (K)	σ(*T*) (K)	*P* (GPa)	σ(P) (GPa)	*V*(NaF) (Å^3)	σ(*V*) (NaF) (Å^3)	*V*(NaF) (cm^3/mol)	σ(*V*) (NaF) (cm^3/mol)
				***VT* Data**			
12	0.5	0.0001	0.000001	97.84299	0.00282	14.73061	0.00043
15	0.5	0.0001	0.000001	97.84666	0.00277	14.73116	0.00042
20	0.5	0.0001	0.000001	97.84709	0.00290	14.73122	0.00044
25	0.5	0.0001	0.000001	97.84485	0.00276	14.73089	0.00042
30	0.5	0.0001	0.000001	97.84136	0.00292	14.73036	0.00044
35	0.5	0.0001	0.000001	97.84738	0.00289	14.73127	0.00043
40	0.5	0.0001	0.000001	97.84565	0.00285	14.73101	0.00043
45	0.5	0.0001	0.000001	97.84318	0.00285	14.73064	0.00043
50	0.5	0.0001	0.000001	97.84659	0.00282	14.73115	0.00042
55	0.5	0.0001	0.000001	97.85396	0.00280	14.73226	0.00042
60	0.5	0.0001	0.000001	97.85864	0.00281	14.73296	0.00042
65	0.5	0.0001	0.000001	97.86610	0.00267	14.73409	0.00040
70	0.5	0.0001	0.000001	97.87720	0.00283	14.73576	0.00043
75	0.5	0.0001	0.000001	97.88756	0.00275	14.73732	0.00041
80	0.5	0.0001	0.000001	97.89896	0.00271	14.73903	0.00041
85	0.5	0.0001	0.000001	97.91380	0.00272	14.74127	0.00041
90	0.5	0.0001	0.000001	97.93067	0.00277	14.74381	0.00042
95	0.5	0.0001	0.000001	97.95184	0.00274	14.74699	0.00041
100	0.5	0.0001	0.000001	97.96060	0.00267	14.74831	0.00040
105	0.5	0.0001	0.000001	97.98514	0.00268	14.75201	0.00040
110	0.5	0.0001	0.000001	98.00916	0.00265	14.75562	0.00040
115	0.5	0.0001	0.000001	98.03709	0.00270	14.75983	0.00041
120	0.5	0.0001	0.000001	98.06282	0.00270	14.76370	0.00041
125	0.5	0.0001	0.000001	98.08669	0.00267	14.76730	0.00040
130	0.5	0.0001	0.000001	98.11125	0.00266	14.77099	0.00040
135	0.5	0.0001	0.000001	98.14459	0.00267	14.77601	0.00040
140	0.5	0.0001	0.000001	98.17008	0.00261	14.77985	0.00039
145	0.5	0.0001	0.000001	98.20415	0.00269	14.78498	0.00040
150	0.5	0.0001	0.000001	98.23781	0.00270	14.79005	0.00041
155	0.5	0.0001	0.000001	98.26473	0.00270	14.79410	0.00041
160	0.5	0.0001	0.000001	98.30273	0.00274	14.79982	0.00041
165	0.5	0.0001	0.000001	98.33555	0.00264	14.80476	0.00040
170	0.5	0.0001	0.000001	98.37664	0.00271	14.81095	0.00041
175	0.5	0.0001	0.000001	98.40535	0.00269	14.81527	0.00040
180	0.5	0.0001	0.000001	98.45253	0.00267	14.82238	0.00040
185	0.5	0.0001	0.000001	98.48216	0.00275	14.82684	0.00041
190	0.5	0.0001	0.000001	98.52525	0.00272	14.83332	0.00041
195	0.5	0.0001	0.000001	98.56162	0.00279	14.83880	0.00042
200	0.5	0.0001	0.000001	98.60464	0.00273	14.84528	0.00041
205	0.5	0.0001	0.000001	98.64644	0.00270	14.85157	0.00041

210	0.5	0.0001	0.000001	98.68428	0.00275	14.85727	0.00041
215	0.5	0.0001	0.000001	98.72179	0.00278	14.86291	0.00042
220	0.5	0.0001	0.000001	98.76533	0.00271	14.86947	0.00041
225	0.5	0.0001	0.000001	98.81052	0.00281	14.87627	0.00042
230	0.5	0.0001	0.000001	98.85389	0.00281	14.88280	0.00042
235	0.5	0.0001	0.000001	98.89705	0.00277	14.88930	0.00042
240	0.5	0.0001	0.000001	98.94190	0.00277	14.89605	0.00042
245	0.5	0.0001	0.000001	98.98415	0.00283	14.90241	0.00043
250	0.5	0.0001	0.000001	99.02599	0.00287	14.90871	0.00043
255	0.5	0.0001	0.000001	99.07564	0.00281	14.91619	0.00042
260	0.5	0.0001	0.000001	99.11644	0.00271	14.92233	0.00041
265	0.5	0.0001	0.000001	99.16885	0.00281	14.93022	0.00042
270	0.5	0.0001	0.000001	99.21078	0.00275	14.93653	0.00041
275	0.5	0.0001	0.000001	99.25670	0.00281	14.94345	0.00042
280	0.5	0.0001	0.000001	99.29596	0.00277	14.94936	0.00042
285	0.5	0.0001	0.000001	99.34873	0.00283	14.95730	0.00043
290	0.5	0.0001	0.000001	99.39377	0.00289	14.96408	0.00044
				***PVT* Data**			
140	1	0.06144	0.00621	98.02054	0.0039	14.75734	0.00059
170	1	0.08002	0.00578	98.20211	0.00438	14.78467	0.00066
200	1	0.16783	0.00555	98.28136	0.00441	14.79660	0.00066
230	1	0.21913	0.00503	98.34312	0.00440	14.80590	0.00066
260	1	0.30260	0.00500	98.44996	0.00443	14.82199	0.00067
290	1	0.33621	0.00515	98.65473	0.00446	14.85282	0.00067
320	1	0.42394	0.00518	98.77968	0.00468	14.87163	0.00070
350	1	0.48356	0.00538	98.94001	0.00479	14.89577	0.00072
350	1	0.69645	0.00900	98.57596	0.00810	14.84096	0.00122
350	1	1.03394	0.01138	97.88881	0.01341	14.73750	0.00202
350	1	1.11817	0.01112	97.65607	0.01065	14.70246	0.00160
140	1	1.00390	0.00811	96.29264	0.00505	14.49720	0.00076
170	1	1.08151	0.00738	96.37965	0.00472	14.51029	0.00071
200	1	1.15191	0.00771	96.44298	0.00488	14.51983	0.00073
230	1	1.17322	0.00773	96.52452	0.00546	14.53211	0.00082
260	1	1.22745	0.00776	96.66693	0.00556	14.55355	0.00084
290	1	1.26773	0.00796	96.87014	0.00531	14.58414	0.00080
320	1	1.31504	0.00856	97.04205	0.00535	14.61002	0.00081
350	1	1.37808	0.00895	97.14335	0.00530	14.62527	0.00080
350	1	1.69109	0.01677	96.44276	0.01093	14.51980	0.00165
350	1	2.19399	0.01828	95.66069	0.01170	14.40205	0.00176
140	1	2.17400	0.01406	94.45156	0.00366	14.22001	0.00055
170	1	2.24908	0.01461	94.49451	0.00382	14.22648	0.00058
200	1	2.28222	0.01500	94.56003	0.00385	14.23635	0.00058
230	1	2.33600	0.01568	94.66267	0.00386	14.25180	0.00058
260	1	2.37572	0.01628	94.77514	0.00392	14.26873	0.00059
290	1	2.36120	0.01632	95.01097	0.00374	14.30424	0.00056
320	1	2.42726	0.01734	95.13767	0.00378	14.32331	0.00057
350	1	2.47148	0.01823	95.27958	0.00414	14.34468	0.00062

350	1	2.93694	0.02709	94.68681	0.01280	14.25543	0.00193
350	1	3.27884	0.03301	93.97327	0.01502	14.14801	0.00226
140	1	3.41406	0.03016	92.78252	0.00617	13.96873	0.00093
170	1	3.43800	0.03074	92.84478	0.00639	13.97811	0.00096
200	1	3.53241	0.03246	92.85623	0.00618	13.97983	0.00093
230	1	3.53457	0.03316	92.95837	0.00640	13.99521	0.00096
260	1	3.55302	0.03411	93.06063	0.00675	14.01061	0.00102
290	1	3.59629	0.03532	93.27512	0.00721	14.04290	0.00108
320	1	3.60011	0.03574	93.40753	0.00686	14.06283	0.00103
350	1	3.66618	0.03758	93.48666	0.00684	14.07475	0.00103
140	1	4.79417	0.05615	91.02293	0.00770	13.70382	0.00116
170	1	4.82798	0.05745	91.06332	0.00808	13.70990	0.00122
200	1	4.92217	0.06025	91.12490	0.00800	13.71918	0.00121
230	1	4.91104	0.06069	91.22318	0.00784	13.73397	0.00118
260	1	4.97649	0.06315	91.29096	0.00812	13.74418	0.00122
290	1	4.93610	0.06324	91.46573	0.00814	13.77049	0.00123
320	1	4.93206	0.06421	91.57248	0.00839	13.78656	0.00126
350	1	5.04667	0.06806	91.63610	0.00888	13.79614	0.00134
295	1	0.00010	0.00001	99.42000	0.001	14.96803	0.00016
				**Liu *et al.*'s Data (Liu *et al.*, 2007)**			
300	2	0.64	0.2	98.596	0.086	14.844	0.013
300	2	0.65	0.2	98.895	0.073	14.889	0.011
300	2	0.60	0.2	98.616	0.020	14.847	0.003
300	2	1.30	0.2	98.025	0.046	14.758	0.007
300	2	1.01	0.2	98.198	0.066	14.784	0.010
300	2	2.19	0.2	96.444	0.093	14.520	0.014
300	2	2.03	0.2	96.258	0.113	14.492	0.017
300	2	4.43	0.2	93.216	0.139	14.034	0.021
300	2	4.59	0.2	92.160	0.046	13.875	0.007
300	2	6.61	0.2	89.709	0.120	13.506	0.018
300	2	6.92	0.2	89.284	0.033	13.442	0.005
300	2	8.54	0.2	87.371	0.073	13.154	0.011
300	2	8.98	0.2	86.979	0.040	13.095	0.006
300	2	10.32	0.2	85.451	0.073	12.865	0.011
300	2	11.08	0.2	84.920	0.020	12.785	0.003
300	2	14.65	0.2	81.991	0.120	12.344	0.018
300	2	17.34	0.2	79.719	0.020	12.002	0.003
300	2	15.38	0.2	81.406	0.159	12.256	0.024
300	2	18.35	0.2	79.314	0.033	11.941	0.005
300	2	16.77	0.2	80.397	0.226	12.104	0.034
300	2	20.18	0.2	77.932	0.013	11.733	0.002
300	2	18.91	0.2	78.623	0.272	11.837	0.041
300	2	22.84	0.2	76.531	0.120	11.522	0.018
300	2	20.78	0.2	77.746	0.272	11.705	0.041
300	2	22.91	0.2	76.929	0.040	11.582	0.006
300	2	27.96	0.2	73.960	1.109	11.135	0.167
300	2	24.11	0.2	76.132	0.100	11.462	0.015

300	2	27.26	0.2	74.432	0.159	11.206	0.024
718	2	21.54	0.2	79.002	0.113	11.894	0.017
885	2	15.94	0.2	83.512	0.146	12.573	0.022
964	2	15.29	0.2	84.375	0.060	12.703	0.009
964	2	17.94	0.2	82.137	0.020	12.366	0.003
978	2	16.63	0.2	83.054	0.020	12.504	0.003
980	2	18.66	0.2	81.572	0.020	12.281	0.003
981	2	20.14	0.2	81.180	0.159	12.222	0.024
981	2	16.89	0.2	82.921	0.093	12.484	0.014
985	2	24.20	0.2	77.707	0.027	11.699	0.004
985	2	22.10	0.2	79.055	0.013	11.902	0.002
985	2	22.05	0.2	79.221	0.013	11.927	0.002
989	2	13.67	0.2	85.651	0.027	12.895	0.004
990	2	15.28	0.2	84.508	0.020	12.723	0.003
989	2	9.66	0.2	90.553	0.040	13.633	0.006
989	2	11.67	0.2	88.048	0.013	13.256	0.002
990	2	12.93	0.2	86.660	0.027	13.047	0.004
990	2	9.23	0.2	90.938	0.013	13.691	0.002
989	2	6.53	0.2	95.574	0.046	14.389	0.007
989	2	6.81	0.2	94.259	0.027	14.191	0.004
989	2	4.6	0.2	98.616	0.066	14.847	0.010
989	2	4.63	0.2	98.470	0.013	14.825	0.002
990	2	2.87	0.2	101.665	0.053	15.306	0.008
989	2	2.73	0.2	101.964	0.020	15.351	0.003

K_s Data

Lewis *et al.* (1967) Data

**T (K)**	**σ(T) (K)**	***P* (GPa)**	**σ(P) (GPa)**	***K_s* (GPa)**	**σ *K_s* (GPa)**
4.2	0.5	0.0001	0.000001	51.4	1.5
300	3.0	0.0001	0.000001	48.5	1.5
**Jones (1976) Data**					
300	3	0.0001	0.000001	48.27	0.3
350	3	0.0001	0.000001	47.38	0.3
400	3	0.0001	0.000001	46.49	0.3
450	3	0.0001	0.000001	45.58	0.3
500	3	0.0001	0.000001	44.67	0.3
550	3	0.0001	0.000001	43.74	0.3
600	3	0.0001	0.000001	42.81	0.3
650	3	0.0001	0.000001	41.86	0.3

Note: Experimental values and their estimated uncertainties are reported to more significant digits than standard practise so as to avoid round-off errors when these data are used for further analysis.

Table S2 Refined EoS parameters for refinement of a model based upon our data alone.

	3rd order BM with MGD model fit to data obtained in this study (*q* refined).
V_0 (cm^3 mol^{-1})	14.9728(5)
K_{0T} (GPa)	45.1(4)
K'_{0T}	6.8(3)
K''_{0T} (GPa^{-1})	−0.3262 (Implied)
θ_D (K)	457(4)
γ	1.500(15)
q	0.9(5)
W-χ^2	2.0635
Scale Factors	1.00 (*VT*), 0.99982(9) (*PVT*)

Table S3 Refined EoS parameters for refinement of a model based upon our data combined with of Liu *et al* (2007).

295 K parameters	3rd order fit to all data with *q* refined	4th order fit to all data with *q* refined	4th order fit to all data with *q* compromise.
V_0 (cm^3 mol^{-1})	14.9720(5)	14.9728(5)	14.9726(5)
K_{0T} (GPa)	48.4(3)	46.1(5)	45.9(5)
K'_{0T}	4.44(10)	6.4(4)	6.6(4)
K''_{0T} (GPa^{-1})	−0.0933 (Implied)	−0.63(11)	−0.70(12)
θ_D (K)	460(4)	458(4)	473(4)
γ	1.591(13)	1.536(15)	1.535(15)
q	1.2(2)	1.2(2)	-
$W-\chi^2$	3.3548	2.7570	2.9007
Scale factors	1.00 (*VT*), 0.99942(10) (*PVT*), 1.0040(13) (Liu)	1.00 (*VT*), 0.99968(11) (*PVT*), 1.0034(12) (Liu)	1.00 (*VT*), 0.99975(11) (*PVT*), 1.0021(10) (Liu)

Table S4 q refined vs q compromise models for NaF EoS from inclusion of all data

295 K Parameters	4th order fit with q refined.	4th order fit with q compromise.
V_0 (cm^3 mol^{-1})	14.9724(5)	14.9722(5)
K_{0T} (GPa)	46.79(14)	46.74(13)
K'_{0T}	5.72(12)	5.83(10)
K''_{0T}(GPa^{-1})	−0.43(4)	−0.46(3)
θ_D (K)	459(3)	474(4)
γ	1.547(11)	1.554(10)
q	0.94(18)	-
W−χ^2	2.73	2.86
Implied α_0 (K^{-1})	9.79(3) × 10^{-5}	9.77(3) × 10^{-5}
Scale Factors	1.00 (*VT*),<br>0.99968(11) (*PVT*),<br>1.0034(12) (Liu),<br>1.00 (Jones),<br>1.00 (Lewis)	1.00 (*VT*),<br>0.99975(11) (*PVT*),<br>1.0021(10) (Liu),<br>1.00 (Jones),<br>1.00 (Lewis)

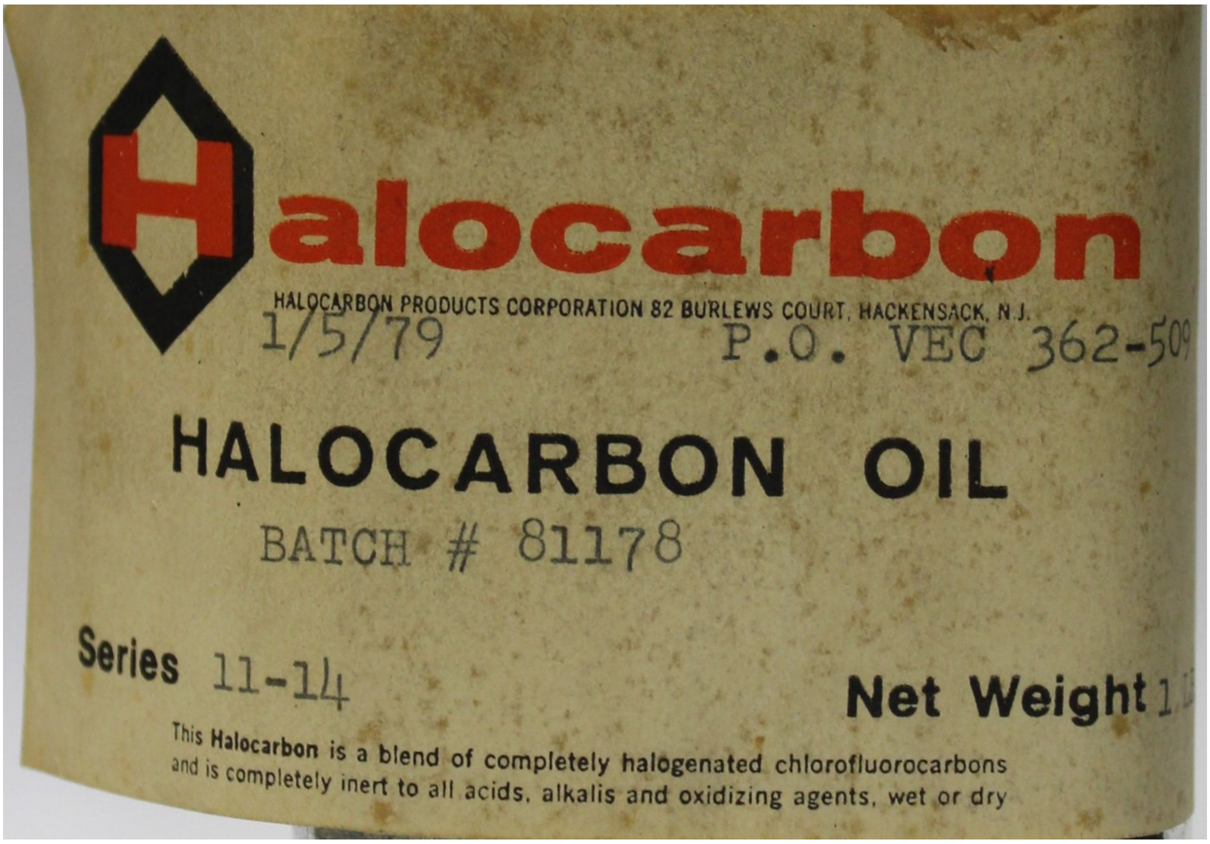

Figure S1 The label on the bottle containing the halocarbon oil used as a pressure transmitting medium in this study.

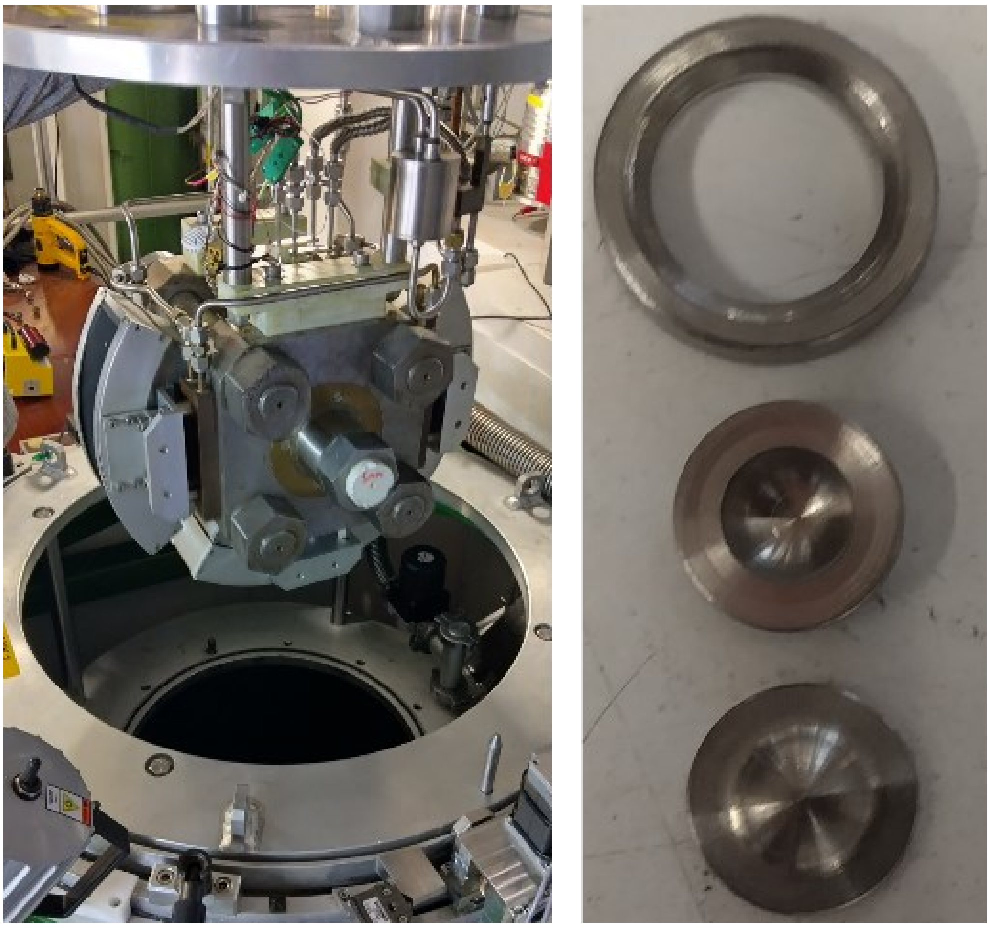

Figure S2 The Paris-Edinburgh press at the PEARL instrument at ISIS (left) and a null scattering TiZr gasket and capsule used to hold sample (right).

References

Jones, L. E. A. (1976). *Phys Earth Planet In* **13**, 105-118

Lewis, J. T., Lehoczky, A. & Briscoe, C. V. (1967). *Phys Rev* **161**, 877-&.

Liu, J., Dubrovinsky, L., Ballaran, T. B. & Crichton, W. (2007). *High Pressure Res* **27**, 483-489.
